# Serotonin and Dopamine Mimic Glucose-Induced Reinforcement in *C. elegans*: Potential Role of NSM Neurons and the Serotonin Subtype 4 Receptor

**DOI:** 10.3389/fphys.2021.783359

**Published:** 2021-12-20

**Authors:** Elizabeth K. C. Schwartz, Eitan N. Sosner, Hayley E. Desmond, Stephanie J. Lum, Ji Ying Sze, Charles V. Mobbs

**Affiliations:** ^1^Fishberg Department of Neuroscience and Friedman Brain Institute, Icahn School of Medicine at Mount Sinai, New York, NY, United States; ^2^Department of Molecular Pharmacology, Albert Einstein College of Medicine, Bronx, NY, United States

**Keywords:** glucose, serotonin, dopamine, reward, obesity, C. elegans

## Abstract

Food produces powerful reinforcement that can lead to overconsumption and likely contributes to the obesity epidemic. The present studies examined molecular mechanisms mediating food-induced reinforcement in the model system *C. elegans*. After a 1-h training session during which food (bacteria) is paired with the odorant butanone, odor preference for butanone robustly increased. Glucose mimicked this effect of bacteria. Glucose-induced odor preference was enhanced similarly by prior food withdrawal or blocking glucose metabolism in the presence of food. Food- and glucose-induced odor preference was mimicked by serotonin signaling through the serotonin type-4 (5-HT4) receptor. Dopamine (thought to act primarily through a D1-like receptor) facilitated, whereas the D2 agonist bromocriptine blocked, food- and glucose-induced odor preference. Furthermore, prior food withdrawal similarly influenced reward produced by serotonin, dopamine, or food, implying post-synaptic enhancement of sensitivity to serotonin and dopamine. These results suggest that glucose metabolism plays a key role in mediating both food-induced reinforcement and enhancement of that reinforcement by prior food withdrawal and implicate serotonergic signaling through 5-HT4 receptor in the re-enforcing properties of food.

## Introduction

Obesity is a leading preventable cause of illness and premature death in the United States and much of the developed world (Jia and Lubetkin, 2010). The obesity epidemic is driven in large part by overconsumption of highly palatable food beyond metabolic requirement (Hill et al., 2003; Finkelstein et al., 2005; Swinburn et al., 2009; Kenny, 2011). Increasingly, it is clear that glucose metabolism plays a major role in the acquisition of the rewarding properties of food, independent of taste, and caloric content (de Araujo et al., 2008; Sclafani and Ackroff, 2012). Since brain reward systems constitute a key component in regulating feeding behavior (Saper et al., 2002; Lutter and Nestler, 2009), understanding the neurobiological circuitry underlying food-induced reward is of considerable interest.

Serotonin and dopamine signaling regulate a range of food-related behaviors including food intake across species. In humans, drugs that increase central serotonergic signaling are approved for weight loss (Weintraub et al., 1984; Hurren and Berlie, 2011), whereas decreasing central serotonergic signaling promotes metabolic syndrome and accumulation of central adiposity (Halder et al., 2007). Serotonin mediates a range of food-related behaviors in *C. elegans*, including activating two feeding motions (pharyngeal pumping and isthmus peristalsis) that regulate intake (Song and Avery, 2012), slowing locomotion, increasing egg-laying rate (Horvitz et al., 1982), and regulating body fat (Srinivasan et al., 2008; Noble et al., 2013). It has been suggested that each of these distinct behaviors is likely mediated by serotonergic signaling *via* independent (yet related) mechanisms or pathways (Noble et al., 2013), with more and more studies supporting this claim. For example, tryptophan hydroxylase (tph-1) functioning in ADF neurons signaling through the serotonin subtype-5 G-protein-coupled receptor (GPCR) regulates pump rate (and thus food intake; Cunningham et al., 2012). Tph-1 signaling through ADF neurons activates the serotonin-gated chloride channel MOD-1 on AIY and AIZ neurons to modulate aversive olfactory learning (Zhang et al., 2005) and on URX body cavity neurons to decrease adiposity (Noble et al., 2013). Although food-induced reward plays a key role in maintaining feeding behavior, the role of serotonin in mediating food-induced reward is not known. The present studies demonstrate a key role for serotonin acting *via* 5-HT4 receptor in food-induced reward.

The dopaminergic system also regulates feeding behavior and food reward – palatable food increases levels of dopamine in the nucleus accumbens (NAc; Hernandez and Hoebel, 1988a,b), and the D2 receptor agonist bromocriptine, recently approved to treat type 2 diabetes, decreases food intake (Thanos et al., 2011) and causes weight loss by unknown mechanisms (Meier et al., 1992). Furthermore, antipsychotic D2 antagonists constitute a major cause of diabetes and obesity in this patient population (American Diabetes Association et al., 2004). Despite the major roles these neurotransmitters play in regulating intake, the mechanisms mediating these phenomena remain unknown.

With a nervous system composed of 302 neurons, a short generation time, and easily observable feeding-related behaviors, the model organism *C. elegans* affords an excellent opportunity to study the mechanisms underlying food-induced reward. As in mammalian neuroendocrine regulatory systems, *C. elegans* feeding behaviors are controlled by serotonin and dopamine signaling (Sze et al., 2000; Hills et al., 2004). Mechanisms regulating energy balance – including those involved in metabolic pathways, adiposity storage and utilization, and insulin signaling – are also conserved (Kimura et al., 1997; Ogg and Ruvkun, 1998; McKay et al., 2003; Morton et al., 2006; Mullaney and Ashrafi, 2009). Therefore the present study used food-induced odor preference (Kauffman et al., 2010) to investigate the role of glucose metabolism, as well as serotonin and dopamine signaling in the development and modulation of food-induced reward.

## Materials and Methods

### General Maintenance and Strains

Strains were maintained as described under standard conditions on nematode growth medium (NGM) with *E. coli OP50* as a food source at 20°C (Brenner, 1974). Animals were developmentally synchronized using the hypochlorite method, and reward assays were carried out when animals reached adult day 1.

Except when noted, strains were provided by the Caenorhabditis elegans Genome Center (CGC). The wild-type strain was N2, and the following mutant strains were used:

CB1370 *daf-2(e1370*; maintained at 15°C), CF1038 *daf-16(mu86)*, GR1321 *tph-1(mg280)*, CX4544 *ocr-2(ak47)*, MT9772 *mod-5(n3314)*, MT1859 *unc-86(n846)*, AQ866 *ser-4(ok512*; https://cgc.umn.edu/strain/AQ866), CB1112 *cat-2(e1112)*, and CB1111 *cat-1(e1111)*.

### Chemotaxis Assay

Chemotaxis assays were performed as previously described (Bargmann et al., 1993). Approximately 100–200 developmentally synchronized adult day 1 animals were placed at the center of a 10 cm plate with butanone (1 μl 1:10 butanone:ethanol) and the control odor ethanol (1 μl) at opposite ends. Sodium azide (1 M; 1 μl) was used to immobilize animals at each odorant. After 1 h, the total number of worms and the number of worms at butanone, ethanol, and the center were counted.

Chemotaxis to butanone was calculated as follows:

$\mathrm{Chemotaxis Index}\;\left(\mathrm{CI}\right)=\left(n_{\mathrm{butanone}}-n_{\mathrm{ethanol}}\right)/\left(\mathrm{total}-n_{\mathrm{origin}}\right)$ (Kauffman et al., 2010).

If all animals chemotax to butanone, the CI would equal 1. If all animals chemotax to ethanol, the CI would equal −1. If equal numbers chemotax to each odorant, the CI would equal 0.

### Reward Assays

Food-induced flavor preference was assessed based on the protocol reported by Kauffman et al. (2010) and Torayama et al. (2007). After three washes, animals were maintained without food in 1.5 ml M9 buffer for 1 h and then transferred to a 6 cm training plate containing OP50 (or the training medium described, such as glucose, serotonin, or dopamine). During training, 2 μl of 1:10 butanone:ethanol was placed on a 2 ml spot of NGM on the lid of the training plate, and the lid was sealed with parafilm. After 1 h of training, animals were transferred to a chemotaxis assay plate where chemotaxis to butanone was tested. Any modifications made to this protocol are described in the text.

### Metabolite and Drug Supplementation

Serotonin hydrochloride (Sigma-Aldrich) was dissolved in M9 buffer and added to each 6 cm plate containing 6.4 ml NGM agar to obtain a final concentration of 6 mM. Dopamine hydrochloride (Sigma-Aldrich) was dissolved in M9 buffer and added to each 6 cm plate containing 10 ml NGM agar to obtain a final concentration of 2 mM. The following were dissolved into NGM agar before solidification in appropriate amounts to yield the final concentrations used: 50 mM glucose (Fisher Scientific), 4 mM 2-deoxyglucose (2DG; Sigma-Aldrich). Bromocriptine (Sigma-Aldrich) was dissolved in 50% ethanol and added to 6.4 ml plates to yield a final concentration of 0.25 mM.

### Statistical Analysis

Data are presented as mean ± SEM. Statistical analyses were performed using GraphPad Prism 5.0a (GraphPad Software, Inc., La Jolla, CA). Differences in learning indices between N2 and mutant strains were compared using a two-tailed t test. Differences in learning indices resulting from different conditions were compared by a two-tailed t test, or by one-way ANOVA followed by the Dunnett *post hoc* test. *p* < 0.05 indicates statistical significance.

## Results

### Food-Induced and Glucose-Induced Odor Preference Can Be Quantified in *C. elegans*

The results described herein are presented in roughly the chronological order in which the studies were carried out.

**Figure 1:**
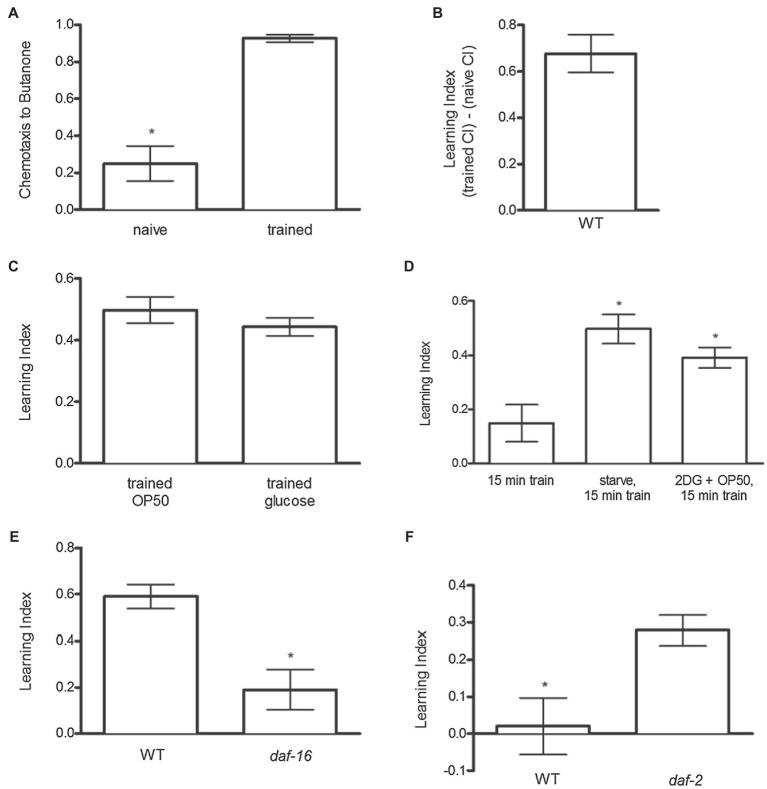
Odor preference development in *C. elegans*. **(A)** During training animals learned to associate a rewarding stimulus (food) with a novel stimulus (butanone). Chemotaxis to butanone was measured before (naïve) and after (trained) training. **(B)** The difference in chemotaxis to butanone before and after training is called the Learning Index (LI); LI = (trained CI) − (naïve CI). **(C)** Glucose increased odor preference similar to OP50. **(D)** Food-induced odor preference was similarly enhanced by preceding training with either a pre-fast or by blocking glucose metabolism with 2DG. **(E)** *daf-16* mutant worms decreased odor preference development after training compared to N2 animals. **(F)** *daf-2* worms increased odor preference development compared to N2 animals after a modified training protocol during which animals were trained for 15 min without 1-h fast pretreatment, so there was as expected no increase in learning index in wild-type animals, but daf-2 mimicked effects of fasting to increase the LI. *p < 0.05 (*t* test). Each column represents at least 4 independent trials, with approximately 50–150 animals tested per trial.

The tendency of *C. elegans* to move toward volatile compounds, such as butanone, can be quantified using the CI (Bargmann et al., 1993). Naïve N2 animals typically exhibited a CI score of ~0.2, demonstrating only a very slight preference for butanone vs. ethanol (Figure 1A). After training – when the odorant butanone is paired with the rewarding stimulus food (OP50 bacteria) – N2 animals increased the CI score to ~0.9 (Figure 1A). The increase in CI from the naïve score is called the learning index (CI_trained_ − CI_naïve_ = LI; Figure 1B) – the LI thus represents the increase in attraction to the odor butanone after it is paired with a reinforcing stimulus. Attraction to butanone typically increased by ~0.6 LI units after training in N2 worms (Figures 1A,B). These results are consistent with those found by Kauffman et al. (2010). Additionally, to assess if glucose could substitute for OP50 to produce odor preference, glucose (50 mM) was paired with butanone during training. Exposure to glucose increased butanone preference to about the same extent as exposure to bacteria (Figure 1C).

### Prolonged Removal of Food or Blocking Glucose Metabolism Similarly Enhances Food-Induced Odor Preference

To assess if nutritional status influences the reinforcing effect of food, the training protocol was slightly modified. N2 animals were trained for 15 min with or without removing food 1 h prior to training. Removal of food for 1 h prior to the 15-min training period significantly enhanced the learning index (Figure 1D). To assess the extent to which this enhancement might be due to reduced glucose metabolism, we assessed if reducing glucose metabolism by the addition of the glycolytic inhibitor 2-deoxyglucose (2DG), in the presence of food, would mimic the enhancing effects of a prior fast on subsequent food-induced reinforcement. Strikingly, inhibiting glucose metabolism in the presence of food prior to training produced the same enhancement of learning index as completely removing food prior to the training period (Figure 1D).

### Effect of Insulin-Like Signaling on Food- and Glucose-Induced Odor Preference

Since insulin-like signaling indicates nutritional abundance and the absence of insulin signaling indicates nutritional deprivation, we assessed whether inactivation of *daf-2*, which encodes a homolog of the mammalian insulin/IGF-1 receptor, and its downstream negative regulator *daf-16, which encodes* a homolog of mammalian FOXO transcription factor (Lin et al., 1997; (Ogg et al., 1997), would influence food-induced odor preference. Kauffman et al. (2010) have previously demonstrated a role of *daf-16* and *daf-2* in association learning. In support of Kauffman et al’s findings (Kauffman et al., 2010), we found that inactivation of *daf-16*, which mediates many effects of DAF-2, reduced food-induced odor preference (Figure 1E). Conversely, when trained for 15 min but without pre-fasting, a condition in which wild-type animals showed no food-induced odor presence, the absence of *daf-2* (mechanistically indicating nutritional insufficiency) increased food-induced odor preference, similar to the effects of food deprivation or inhibition of glucose metabolism (Figure 1F).

### Serotonin Released From the NSM Neurons and Signaling Through the 5-HT4 Receptor Modulates Food-Induced Reinforcement

**Figure 2:**
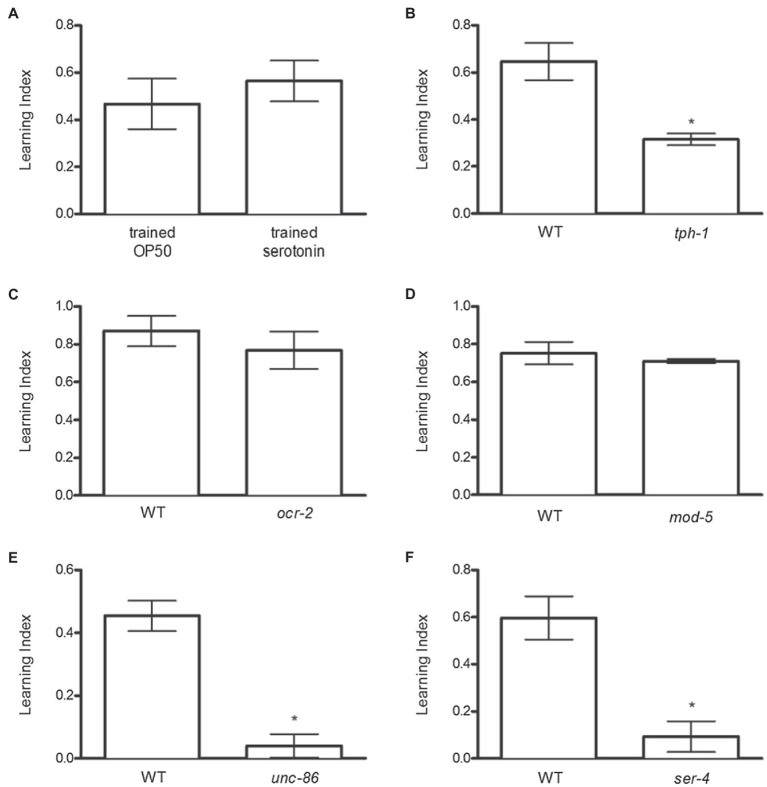
Serotonin (released from NSM neurons) signaling through 5-HT4 receptor increases food-induced odor preference. **(A)** Serotonin increased odor preference similar to OP50. **(B)** *tph-1* mutant animals exhibited deficits in odor preference development. **(C)** *ocr-2* and **(D)** *mod-5* mutant animals increased odor preference, similar to N2 animals. **(E)** *unc-86* mutant animals displayed deficits in food-induced odor preference development. **(F)** *ser-4* mutant animals exhibited deficits in odor preference compared to N2 animals. *p < 0.05 (*t* test). Each column represents at least 3 independent trials, using approximately 50–150 animals per trial.

Because serotonin regulates behaviors relevant to feeding across species and signals the presence of food in *C. elegans*, we tested whether exogenous serotonin could mimic the effects of OP50 to promote odor preference. Serotonin increased odor preference similarly to OP50 (Figure 2A). Conversely, genetic inactivation of *tph-1*, which encodes tryptophan hydroxylase, catalyzing the first and rate-limiting step in serotonin synthesis (Sze et al., 2000), diminished food-induced odor preference (Figure 2B).

The next studies assessed which serotonergic neurons are required for food-induced odor preference. In *C. elegans*, serotonin is present in four classes of bilaterally symmetrical neurons – the NSM pharyngeal secretory neurons, the ADF chemosensory neurons, the HSN egg-laying neurons, and the AIM interneurons – as well as in the single RIH interneuron. Because *tph-1* expression is controlled by cell-specific mechanisms in different neurons (Sze et al., 2002), strains carrying mutations in neuron-specific regulators of *tph-1* can be utilized to indicate which neurons may be responsible for producing serotonin necessary for food-induced odor preference. Mutation of the transient receptor potential V (TRPV) ion channel gene *ocr-2* dramatically down-regulates *tph-1* expression in the ADF neurons (Zhang et al., 2004). However, *ocr-2* mutant animals increased odor preference similarly to N2 animals, indicating that serotonin produced in ADF neurons is not necessary for food-induced odor preference (Figure 2C). Unlike NSM, ADF, and HSN neurons, the AIM and RIH interneurons only take up extracellular serotonin – they do not synthesize it (Kullyev et al., 2010; Jafari et al., 2011). The gene *mod-5* encodes the serotonin reuptake transporter (SERT) necessary for extracellular serotonin reuptake into neurons (Ranganathan et al., 2001) – consequently, *mod-5* mutant animals exhibit serotonin immunoreactivity in NSM, ADF, and HSN neurons only and not in AIM or RIH interneurons. The observation that *mod-5* mutant animals exhibited food-induced odor preference similarly to N2 animals (Figure 2D) demonstrated that serotonin uptake by RIH and AIM is not necessary for food-induced odor preference. Finally, null mutations in the POU-domain transcription factor *unc-86* decrease *tph-1* expression in NSM and HSN but not ADF neurons (Sze et al., 2002). We found that genetic inactivation of *unc-86* blocked food-induced odor preference (Figure 2E). Taken together, these results implicated NSM as the neuron necessary for producing serotonin involved in food-induced odor preference.

To determine the serotonin receptor subtype necessary for mediating serotonin’s rewarding effects, we screened the serotonin GPCR KO strains (*ser-1*, *ser-4*, *ser-5*, and *ser-7*; data not shown) for deficits in odor preference development. Only genetic ablation of ser-4 decreased food-induced odor preference (Figure 2F).

### Dopamine Increases, While the D2 Dopamine Agonist Bromocriptine Decreases, Food-Induced Odor Preference

**Figure 3:**
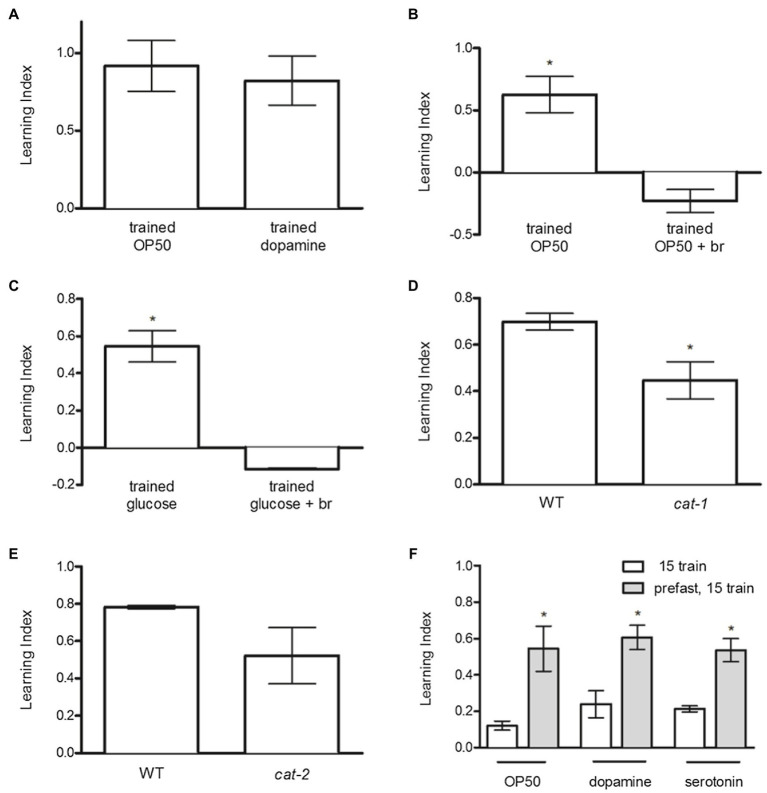
Dopamine D1- and D2-like receptor signaling antagonistically regulates food and glucose-induced odor preference. **(A)** Exogenous DA increased odor preference similar to OP50. **(B)** Bromocriptine blocked odor preference induced by OP50 and **(C)** glucose. **(D)** *cat-1* mutant worms exhibited decreased learning index compared to N2 animals. **(E)** The learning indices of *cat-2* mutants and N2 animals were similar. **(F)** Fasting before training increased odor preference produced by food, dopamine, and serotonin similarly. *p < 0.05 (*t* test). Each column represents at least 3 independent trials, using approximately 50–150 animals per trial.

To investigate the hypothesis that D1- and D2-like dopamine receptors are antagonistic in food-induced reward, we assessed the effects of exogenous dopamine (DA; thought to act mainly through D1-like receptor activity; Missale et al., 1998) and the D2 agonist bromocriptine, on odor preference. DA produced odor preference similar to OP50 (Figure 3A), whereas bromocriptine completely blocked odor preference induced by OP50 (Figure 3B) or glucose (Figure 3C).

### Reduction of Synaptic Serotonin and Dopamine by Ablation of the Vesicular Monoamine Transporter Reduces Food-Induced Odor Preference, While Reduction of Dopamine Alone by Ablation of Tyrosine Hydroxylase Is Not Sufficient to Block Food-Induced Odor Preference

CAT-1 encodes the vesicular monoamine transporter utilized in uptake of dopamine and serotonin (Sulston et al., 1975), and *cat-1* mutants have been shown to have reduced synaptic dopamine and serotonin (Duerr et al., 1999). It is therefore of some interest that genetic inactivation of *cat-1* reduces food-induced odor preference (Figure 3D).

CAT-2 encodes tyrosine hydroxylase, the enzyme that catalyzes the rate-limiting step in catecholamine synthesis (Sawin et al., 2000). The mutation of *cat-2* used in this study does not reduce serotonin but reduces dopamine levels by 75% (Yao et al., 2010). The effect of the *cat-2* mutation on food-induced odor preference was not statistically significant, but the trend to reduce preference was clearly apparent (Figure 3E).

### Food Deprivation Similarly Enhances Food-, Dopamine-, and Serotonin-Induced Odor Preference

To assess if nutritional deprivation would influence food-induced odor preference, we used a sub-threshold 15-min training period paired with OP50, dopamine, or serotonin, after a 1-h period in the presence or absence of food. Nutritional deprivation enhanced odor preference produced by OP50, serotonin, or dopamine similarly (Figure 3F).

## Discussion

Brain reward systems activated by highly palatable foods are major regulators of feeding behavior (Saper et al., 2002; Lutter and Nestler, 2009; Zheng et al., 2009; Shomaker et al., 2010; Kenny, 2011). Given the growing obesity and type 2 diabetes epidemics, the mechanisms by which reward from highly reinforcing foods – including glucose – increase consumption is of considerable interest. Furthermore, although both serotonin and dopamine play a role in neuroendocrine signaling and regulate feeding behaviors across species, the underlying molecular mechanisms remain unknown. This paper reports several novel observations regarding molecular mechanisms underlying the re-enforcing effects of food in *C. elegans*. First, we demonstrate for the first time that glucose is rewarding to *C. elegans*, based on the rapid acquisition of preference of a neutral odor when paired with glucose. Second, we demonstrate that serotonin mimics glucose and food-induced reward, acting through 5-HT4 receptor. We also demonstrate for the first time that dopamine also mimics reward produced by glucose and that the D2 receptor agonist reduces this reward. Of particular interest we report that both food withdrawal and antagonism of glucose metabolism prior to pairing enhance food-induced reward.

Not all food sources signal similarly as nutrient signals. Foods high in sugar are particularly reinforcing (Berthoud et al., 2011), and glucose is particularly rewarding compared to other sugars, such as fructose, as well as other nutrient sources, such as the amino acid serine (Ren et al., 2010; Sclafani and Ackroff, 2012). Studies completed in our lab (data not shown) have demonstrated that similarly to glucose the amino acid leucine increases chemotaxis after training, whereas serine does not, demonstrating that, as in mammals, in *C. elegans* not all nutrients are equally reinforcing. Here we demonstrated that novel finding that glucose increases odor preference similar to OP50, supporting the hypothesis that the reinforcing effects of glucose are conserved across species.

Hunger increases reinforcing effects of food (i.e., “hunger is the best sauce”). For example, fasting increases ventral striatum activation after consumption of highly palatable food in humans (Goldstone et al., 2009). Similarly, nutritional deprivation enhanced food-induced odor preference in *C. elegans* (Figure 1D). 2DG induces hunger in humans (Thompson and Campbell, 1977) and other mammals (Bergen et al., 1996), possibly by inhibiting neuroendocrine sensing of glucose. Similarly, in *C. elegans* 2DG in the presence of OP50 enhanced food-induced odor preference (Figure 1D) similar to nutritional deprivation, indicating that glucose sensing also plays a role in mediating effects of nutritional state on reinforcing properties of food.

When nutritional resources are abundant, insulin-like ligands signaling through DAF-2 leads to DAF-16 phosphorylation and its consequent sequestration in the cytoplasm (Henderson and Johnson, 2001; Lee et al., 2001; Lin et al., 2001). Conversely, when food is scarce, DAF-2 signaling decreases, leading to DAF-16 translocation to the nucleus and subsequent gene expression (Ogg et al., 1997; Honda and Honda, 1999; Henderson and Johnson, 2001; Garsin et al., 2003; Libina et al., 2003). Since genetic inactivation of *daf-2* enhanced, whereas inhibition of *daf-16* inhibited, food-induced odor preference, we conclude that reduced activity of insulin-like signaling mediates at least some of the effect of nutritional deprivation to increase the reinforcing properties of food (Figures 1E,F). These findings support those previously demonstrated by Kauffman et al. (2010); however in contrast to these authors’ findings that *daf-2* and WT animals learn at a similar rate when trained after a fast, here we show that in the absence of fasting prior to training, *daf-2* mutants exhibit enhanced odor preference development compared to WT animals. This finding indicates that the *daf-2* mutation mimics starvation – this effect of enhanced odor preference development compared to WT animals is perhaps then masked when both groups are fasted prior to conditioning.

Increasing central serotonergic signaling promotes satiety and weight loss in humans (Weintraub et al., 1984; Hurren and Berlie, 2011) and non-human mammals (Edwards and Stevens, 1991; Vickers and Dourish, 2004), whereas impaired serotonin signaling causes hyperphagia and obesity in a 5-HT_2c_ knockout mouse model (Tecott et al., 1995). In *C. elegans*, serotonin is known to signal the presence of food – similarly to being exposed to the worm food source OP50 bacteria, exogenous serotonin slows locomotion and increases pump and egg-laying rate (Horvitz et al., 1982; Srinivasan et al., 2008). Interestingly, *tph-1* expression only in ADF neurons supports aversive but not attractive learning, while *tph-1* expression in both NSM and ADF neurons supports both aversive and attractive learning, suggesting that NSM neurons play a role in “evaluating attractive components of food-related signals” (Zhang et al., 2005).

Our data indicate a role for serotonin released from NSM neurons in food-induced odor preference (Figure 2). Anatomically, NSM neurons are well suited to convey the presence of nutritional resources through neuroendocrine signaling. NSM neuron sensory endings extend into the pharyngeal lumen and NSM axons extend into the pseudocoelom where released molecules can signal in an endocrine fashion (Albertson and Thomson, 1976; White et al., 1986; Altun and Hall, 2009). Furthermore, NSM neurons are secretory and modulate feeding behavior (Avery and Horvitz, 1989). Since genetic inactivation of *ocr-2*, which blocks serotonin synthesis in ADF neurons, and inactivation of *mod-5*, which blocks serotonin reuptake in AIM and RIH neurons, fail to block food-induced odor preference, whereas inactivation of *unc-86*, which eliminates serotonin synthesis in NSM and HSN neurons, blocks food-induced odor preference, these data are consistent with the hypothesis that food-induced odor preference may be mediated by serotonin released from NSM neurons (Figures 2D–F). Furthermore, 5-HT4 receptor appears to be the key receptor mediating these effects of serotonin (Figure 2F). More studies utilizing cell-specific reconstitution are needed to validate these hypotheses.

Dopamine is also a key regulator of feeding behavior (Hernandez and Hoebel, 1988a,b). In mice, ablation of tyrosine hydroxylase and thus dopamine produces deficits in both motor output and motivated behavior, and animals die due to failure to initiate feeding (Zhou and Palmiter, 1995; Szczypka et al., 2000). Delivery of the TH gene primarily to the ventral striatum, which regulates reward, rescues lethality and restores feeding without improving motor deficits (Szczypka et al., 1999). Conversely, the dopamine D2 receptor agonist bromocriptine, approved for use to treat diabetes because of its ability to reduce hyperglycemia in individuals with type 2 diabetes mellitus (Barnett et al., 1980), decreases adiposity in both humans (Meier et al., 1992) and other mammals (Cincotta et al., 1991, 1997). As in mammalian systems, in *C. elegans* dopamine acts through D1- and D2-like receptors that signal through orthologs of the major mammalian G-proteins (Gaq and Gao, respectively) to direct locomotion (Jansen et al., 1999; Suo et al., 2002; Chase et al., 2004; Allen et al., 2011). Since exogenous dopamine, like OP50, increased odor preference, while exogenous bromocriptine blocked both food- and glucose-induced odor preference (Figures 3A–C), we conclude that D1- and D2-like signaling antagonistically drive food-induced reward, with predominant activation of lower affinity D1-like receptors enhancing reward when dopamine levels are high, and predominant activation of higher affinity D2-like receptors decreasing reward when dopamine levels are low (Avery and Horvitz, 1989; Missale et al., 1998; Marcellino et al., 2012).

Genetic inactivation of *cat-1*, which decreases synaptic dopamine and serotonin function (Sulston et al., 1975; Duerr et al., 1999), inhibited food-induced odor preference, whereas partial inactivation of *cat-2*, leading to deficiency in dopamine alone, failed to inhibit food-induced reward statistically, although clearly there was a trend toward reduced reward (Figures 3D,E). These observations support that serotonin and dopamine signaling function in parallel to mediate food-induced odor preference. Furthermore, since nutrition deprivation enhanced odor preference produced by food, dopamine, and serotonin similarly, we hypothesize that nutrition deprivation enhances learned odor preference through a post-synaptic mechanism (Figure 3F). However, this hypothesis is subject to the caveat that the synaptic localizations of these receptors have not been definitively demonstrated in *C. elegans*.

Extending previous studies (Tsalik and Hobert, 2003; Luedtke et al., 2010), these results are consistent with a basic model mediating food reward in *C. elegans*. In the presence of an odor, AWC neurons are inactive, and signaling by downstream interneurons increases the dwell time near a food source (Chalasani et al., 2007). Also during exposure to food, we hypothesize that food- or glucose- sensing causes NSM neurons to humorally release serotonin, which signals through 5-HT4 receptor expressed on the RIB interneuron (Tsalik et al., 2003), in turn acting on AVA neurons to modulate ventral motor neurons, leading to forward and backward movement keeping the animal close to the odor or food source (future cell-specific studies would be necessary to validate this proposed circuit). The D1-like receptor is also expressed on the RIB interneuron (Sanyal et al., 2004), plausibly enhancing the activity of this neuron by dopamine. When an odorant is removed, AWC neurons become activated leading to downstream ventral motor signaling that induces turning behavior (Chalasani et al., 2007), which is a main feature of local area search behavior. Removal of a food source would likely correspond to odorant removal – during this time we suggest that hunger enhances activity at 5-HT4 receptor and D1-like receptors on the RIB interneuron (further cell-specific studies would be necessary to validate this hypothesis). In mammals, D1- and D2-like receptors are in different populations of neurons in the nucleus accumbens. D2-like receptors are present on the RIC interneuron (Suo et al., 2009). RIC interneurons receive input from CEP mechanosensory neurons concerning the presence of bacteria (White et al., 1986; Sawin et al., 2000) and synapse on to the AVA command interneuron which modulates ventral motor neurons. Alternatively, CEP may signal through the D2-like receptor expressing SIA interneuron (Suo et al., 2009), which lacks synaptic output but does form gap junctions with the RIB interneuron.

Food-induced reward is regulated by a complex interaction of environmental inputs, such as nutrient availability, and internal inputs, such as nutrient selection, current nutritional state, neuroendocrine signaling, and prior experience (Zheng and Berthoud, 2008). Determining how serotonin and dopamine receptor signaling regulate food-induced reinforcement is more tractable in a nervous system containing 302 neurons than in mammalian neuronal systems. Investigating this circuitry in *C. elegans* affords an opportunity to study a simple form of food reward learning that is easily observable on the level of the whole organism. For example, genes regulating food-induced reward may be identified by high-throughput RNAi screens. Because of the known conservation of the many pathways regulating adiposity and food intake between mammals and *C. elegans*, studies such as these can lead to better, and more specific, therapeutic interventions that modulate food-induced reward.

While the present studies are informative, a number of caveats are pertinent to the interpretation of these results, and future studies should clarify these caveats. For example, future studies would carry out more extensive dose–response relationships to allow more precise interpretation of the likely targets mediating these effects. Similarly, while the present studies clearly demonstrate a role for dopamine in mediating reinforcing effects of food and glucose in *C. elegans*, future studies using strains with genetic ablation of specific dopamine receptors (e.g., assessing effects of food and glucose-induced reward in dop-1, dop-2, dop-3, or mod-1 mutant strains, which might reveal a possible involvement of distinct dopamine receptor subtypes). Similarly, the present studies are based on efficacy of manipulations to increase preference for butanone, although butanone is already somewhat reinforcing compared to ethanol. Future studies might examine efficacy of these reinforcing effects on more neutral stimuli which have no reinforcing properties. Other future studies might examine effects of more specific agonists or antagonists of the *C. elegans* 5-HT4 receptor to corroborate the genetic studies reported here.

## Data Availability Statement

The raw data supporting the conclusions of this article will be made available by the authors, without undue reservation.

## Author Contributions

ES designed, oversaw and carried out most of the studies, and wrote the manuscript. HD and SL assisted with some studies. JS contributed some *C. elegans* strains and provided advice. CM designed the studies and wrote the manuscript. All authors contributed to the article and approved the submitted version.

## Funding

ES was supported by the American Diabetes Association Clinical Scientist Predoctoral Training Award and the Ruth L. Kirschstein National Research Service Award for Individual Predoctoral Fellows. Studies funded by NIDDK.

## Conflict of Interest

The authors declare that the research was conducted in the absence of any commercial or financial relationships that could be construed as a potential conflict of interest.

## Publisher’s Note

All claims expressed in this article are solely those of the authors and do not necessarily represent those of their affiliated organizations, or those of the publisher, the editors and the reviewers. Any product that may be evaluated in this article, or claim that may be made by its manufacturer, is not guaranteed or endorsed by the publisher.
